# Epilepsy Seizures in Spontaneously Hypertensive Rats After Acoustic Stimulation: Role of Renin–Angiotensin System

**DOI:** 10.3389/fnins.2020.588477

**Published:** 2020-12-23

**Authors:** Christiane Becari, Giorgia Lemes Pereira, José A. C. Oliveira, Katarzyna Polonis, Norberto Garcia-Cairasco, Claudio M. Costa-Neto, Marilia G. A. G. Pereira

**Affiliations:** ^1^Division of Vascular and Endovascular Surgery, Department of Surgery and Anatomy, Ribeirão Preto Medical School, University of São Paulo, Ribeirão Preto, Brazil; ^2^Department of Biochemistry, Biomedical Sciences Institute, Federal University of Alfenas, Alfenas, Brazil; ^3^Department of Physiology, Ribeirão Preto Medical School, University of São Paulo, Ribeirão Preto, Brazil; ^4^Department of Laboratory Medicine and Pathology, Mayo Clinic, Rochester, MN, United States; ^5^Department of Biochemistry and Immunology, Ribeirão Preto Medical School, University of São Paulo, Ribeirão Preto, Brazil

**Keywords:** hypertension, epilepsy, renin–angiotensin system, ACE inhibitors, AT1 antagonist, SHR

## Abstract

Hypertension is a common comorbidity observed in individuals with epilepsy. Growing evidence suggests that lower blood pressure is associated with reduced frequency and severity of seizures. In this study, we sought to investigate whether the renin–angiotensin system (RAS), which is a critical regulator of blood pressure, is involved in the pathogenesis of audiogenic epilepsy-related seizures in a hypertensive rat model. Spontaneously hypertensive rats (SHRs) were given RAS inhibitors, angiotensin-converting enzyme (ACE) inhibitor or angiotensin II type I receptor (AT1R) antagonist, for 7 days prior to inducing epileptic seizures by acoustic stimulation. After the pretreatment phase, blood pressure (BP) of SHRs normalized as expected, and there was no difference in systolic and diastolic BP between the pretreated SHRs and normotensive rat group (Wistar). Next, treated and untreated SHRs (a high BP control) were individually subjected to acoustic stimuli twice a day for 2 weeks. The severity of tonic–clonic seizures and the severity of temporal lobe epilepsy seizures (product of forebrain recruitment) were evaluated by the mesencephalic severity index (Rossetti et al. scale) and the limbic index (Racine’s scale), respectively. Seizures were observed in both untreated (a high BP control) SHRs and in SHRs treated with AT1R antagonist and ACE inhibitor. There was no statistical difference in the mesencephalic severity and limbic index between these groups. Our results demonstrate that SHRs present seizure susceptibility with acoustic stimulation. Moreover, although RAS inhibitors effectively reduce blood pressure in SHR, they do not prevent developing epileptic seizures upon acoustic stimulation in SHR. In conclusion, our study shows that RAS is an unlikely link between hypertension and susceptibility to epileptic seizures induced by acoustic stimulation in SHRs, which is in contrast with the anticonvulsant effect of losartan in other animal models of epilepsy.

## Introduction

Epilepsy, affecting 50 million people worldwide (World Health Organization, 2019), is a chronic neurological disorder characterized by unprovoked recurrent seizures more than 24 h apart (Fisher et al., 2014). Individuals with epilepsy are reported to have a higher prevalence of psychiatric and cardiovascular comorbidities (Keezer et al., 2016). A prevalence of heart disease in adult patients with a history of epilepsy is reported to be about 9% higher than in individuals without a history of epilepsy (Zack and Luncheon, 2018). Interestingly, hypertension and hyperlipidemia, two classic cardiovascular risk factors, are more prevalent than a psychiatric diagnosis in epileptic adult populations (Wilner et al., 2014). This suggests that high blood pressure may be associated with cardiovascular events and sudden death in patients with epilepsy (Szczurkowska et al., 2019). Conversely, sudden deaths observed in this group of patients may be partially related to cardiovascular abnormalities (Ngugi et al., 2013).

Renin–angiotensin system (RAS), a hormone complex involved in blood pressure control, regulates cardiovascular functions; however, when dysregulated it contributes to pathological processes leading to hypertension and cardiovascular disease (de Gasparo et al., 2000; Bader and Ganten, 2008; Becari et al., 2011, 2017). Classically, RAS is described as a cascade in which angiotensinogen (AGT) is cleaved by renin to circulating angiotensinogen-forming angiotensin I (Ang I) and next to angiotensin II (Ang II) by angiotensin-converting enzyme (ACE). AngII, the strongest effector of RAS, exerts effects by activating angiotensin 1 (AT1Rs) and angiotensin 2 (AT2R) receptors. It is widely recognized that RAS components are also expressed in other organs and tissues such as the brain. Local RAS in the central nervous system has been shown to be involved (directly or indirectly) in the modulation of some cognitive functions such as memory and learning (Farag et al., 2017; Huber et al., 2017; Mascolo et al., 2017).

The spontaneously hypertensive rat (SHR) is a classical hypertension model (Okamoto and Aoki, 1963) and also has been considered to model other diseases such as attention-deficit hyperactivity disorder (ADHD) with over activity, impulsiveness, deficient sustained attention, depression (Tchekalarova et al., 2011), and more recently epilepsy (Vorobyov et al., 2011; Tchekalarova et al., 2014, 2015, 2016; Russo et al., 2017; Atanasova et al., 2018). The first description of SHR presenting kindling with fewer electroencephalogram (EEG) after discharges was by amygdala or/and pyriform cortex stimulation (Greenwood et al., 1989). Other studies have shown that SHR are susceptible to epilepsy induced by kainate (Tchekalarova et al., 2016), as well as by pilocarpine (Mello et al., 1993; Scorza et al., 2005; Grosser et al., 2020). However, the response of SHR to acoustic stimulation with audiogenic seizures has not been shown.

Our group has shown previously that the RAS system is involved in epileptogenesis in the Wistar audiogenic rat (WAR) experimental model (Garcia-Cairasco et al., 2017). Treatment with RAS inhibitors not only lowered blood pressure levels but importantly reduced the severity of tonic–clonic seizures and blocked limbic seizures during sound stimulation (audiogenic kindling) (Pereira et al., 2010). We also demonstrated that both a local non-classical pathway was involved in Ang1–7 generation in the hippocampus of rats, suggesting a functional relevance of local RAS in the pathophysiology of epilepsy (Pereira et al., 2013). Although WARs have been genetically developed initially for the epilepsy phenotype, they display, in addition to neuropsychiatric comorbidities (Garcia-Cairasco et al., 2017), systemic cardiovascular alterations (Fazan et al., 2015) and impaired central respiratory chemoreflex (Totola et al., 2017). Other groups have shown RAS contribution to epilepsy using animal models such as the kainate (KA) model of temporal lobe epilepsy (TLE) and the pilocarpine in Wistar and SHR (Mello et al., 1993; Scorza et al., 2005; Ivanova et al., 2015; Tchekalarova et al., 2016; Atanasova et al., 2018; Grosser et al., 2020). Interestingly, the long-term treatment with losartan after kainate (KA)-induced *status epilepticus* (SE) exerted a disease-modifying effect on spontaneous seizure activity and neuronal damage in a SHR (Tchekalarova et al., 2014, 2015, 2016). Interestingly, losartan and enalapril treatment in SHR submitted to acoustic stimulation with audiogenic seizures has not been investigated yet.

Based on evidence presented above, we hypothesized that the elevated blood pressure, via RAS, is involved in the development of epilepsy induced by acoustic stimulation and that a pretreatment with RAS inhibitors would reduce epileptic seizures in SHR.

## Materials and Methods

### Animals

Experiments were conducted on 9–12 week old male SHR and Wistar rats. Animals were maintained in a controlled environment with a constant 12:12 h light–dark cycle and provided with food and water *ad libitum*. All surgical procedures were performed under tribromoethanol anesthesia (250 mg/kg, ip). All experiments were conducted following the National Institutes of Health Guide for the Care and Use of Laboratory Animals. Every effort was done in order to avoid unnecessary suffering and stress to the animals.

### Protocol Design

In the first protocol group, blood pressure measurements, acoustic stimulation, and seizure evaluation were performed in SHR and in Wistar rats as a control. The aim of this protocol was to determine whether SHR would develop epileptic seizures when submitted to acoustic stimulation following behavioral evaluation protocols by Garcia-Cairasco et al. (1992) and Pereira et al. (2010), developed for genetically developed Wistar audiogenic rat (WAR) strain (Doretto et al., 2003). The acoustic stimulation and seizure evaluation were described ahead.

In the second protocol group, blood pressure measurements were performed before the treatment initiation and, after 7 days of enalapril treatment (Galena Company), losartan treatment (Galena Company) or vehicle treatment (water) in SHR and in Wistar rats as a control, as described by Pereira et al. (2010). The aim of this protocol was to analyze whether targeting RAS pathway attenuates epileptic seizures induced by acoustic stimulation.

### Systolic Blood Pressure Measurements

Systolic blood pressure (SBP) was measured using a tail-cuff plethysmography method (MK-II; E&M Instrument) in conscious SHR and Wistar rats in a prewarmed (10 min at 38°C) and thermostatically controlled heating cage box. In the first protocol group, blood pressure measurements were performed before the start of acoustic stimulation in Wistar rats and in SHRs. In the second protocol group, blood pressure measurements were performed before the start of the treatment and after 7 days of a given treatment. Final SBP values were obtained by averaging three successful consecutive readings.

### Acoustic Stimulation and Seizure Evaluation

Acoustic stimulation was performed twice a day (kindling protocol as described by Pereira et al., 2010) at fixed times (between 08:00–09:00 h and 16:00–17:00 h) during 10 consecutive days after 7 days of pretreatment with enalapril, losartan, or vehicle. An acoustically isolated box was used to expose each animal to a high-intensity sound, until tonic seizure appearance or for a maximum time of 1 min (Pereira et al., 2010). The sound of a ringing bell (120 dB) was digitized with a high-pass filter (N500 Hz) and reproduced with a personal computer coupled to amplifiers and tweeters under the top of the cage. Rat’s behavior was recorded during the stimulus duration and for 1 min after exposure. The severity of tonic–clonic seizures was evaluated using the mesencephalic severity index (Rossetti et al., 2006). Racine’s scale (limbic index) was used to assess the severity of temporal lobe epileptic seizures (Racine, 1972; Galvis-Alonso et al., 2004). Repeated audiogenic seizures were named audiogenic kindling by Marescaux et al. (1987), and we reproduced this phenomenon in WARs (Garcia-Cairasco et al., 1996a; Dutra Moraes et al., 2000; Pereira et al., 2010). Briefly, development of chronic seizures is characterized by an increase in the mesencephalic index, followed by a decrease in the mesencephalic index values, and a concomitant increase in limbic index values. Observed changes are due to the recruitment of different brain regions, mostly prosencephalic and limbic, which leads to different behavioral alterations in animals with epileptic background (Garcia-Cairasco et al., 1996b, 2017; Dutra Moraes et al., 2000; Romcy-Pereira and Garcia-Cairasco, 2003; Pereira et al., 2010). The treatment groups were subjected to induction of audiogenic seizures (Garcia-Cairasco et al., 1992), and data were collected in a blind manner.

### Pharmacological Treatment

SHRs were pretreated by gavage with ACE inhibitor (enalapril, 10 mg kg^–1^ of body weight⋅day^–1^), AT_1_R antagonist (losartan, 50 mg kg^–1^ of body weight⋅day^–1^), and vehicle once a day (between 12:00 and 14:00 h) for 7 days prior to acoustic stimulation and for 10 consecutive days during acoustic stimulation. The efficacy of the doses was validated *in vivo* in SHRs, such as both enalapril and losartan showed a significant antihypertensive effect (reduction of approximately 30% in mean BP).

### Statistical Analyses

Data are presented as mean ± SEM of the indicated number of independent experiments. Statistical analyses were done using one-way analyses of variance (ANOVAs) or unpaired Student’s *t*-tests, as appropriate. *P* < 0.05 were considered as statistically significant. Statistical analysis and graph plotting were performed in GraphPad Prism software program (San Diego, CA, United States).

## Results

**FIGURE 1:**
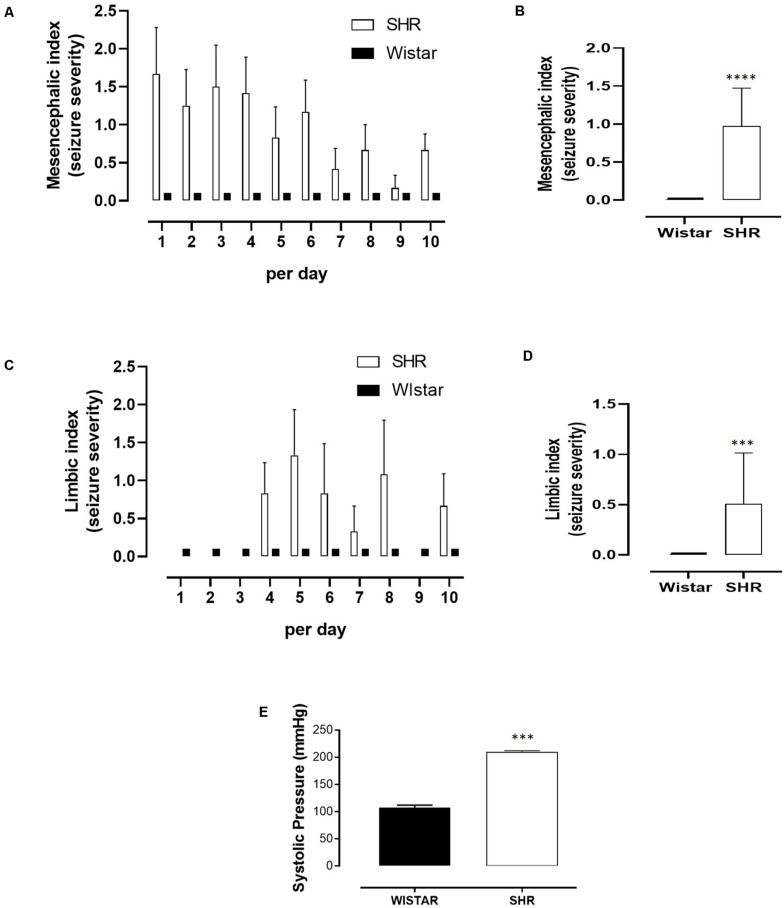
Seizure severity during acoustic stimulation and systolic blood pressure (SBP) measure comparing Wistar rats and spontaneous hypertensive rats (SHR). **(A)** Severity indexes for tonic–clonic seizures (mesencephalic index) per day. **(B)** Mesencephalic indexes mean for tonic–clonic seizures. **(C)** Severity indexes for temporal lobe epilepsy seizures (limbic index) per day. **(D)** Limbic indexes mean for temporal lobe epilepsy seizures. **(E)** Systolic pressure of Wistar and SHR rats measured prior to the first acoustic stimulus. **(A,C)** Data are expressed as mean ± SEM for each group (Wistar or SHR), per day. **(B,D,E)** Data are expressed as mean ± SEM for each group (Wistar or SHR), and Student’s *t*-test was used to compare between groups. ***p < 0.001. ****p < 0.0001. For acoustic stimulation, Wistar (*n* = 10) and SHR (*n* = 10). For SBP measure, Wistar (*n* = 15) and SHR (*n* = 23).

First, we demonstrated that SHR showed seizure susceptibility with acoustic stimulation. The seizure severity was described based on indexes recorded for tonic–clonic seizures (mesencephalic index, Figures 1A,B) and limbic seizures (limbic index, Figures 1C,D). The first day of the audiogenic kindling protocol of SHR presented seizure behavior characterized by wild running and jumping (mesencephalic index equal 2) and, after day 4, the appearance of limbic behavior such as myoclonus (limbic index equal 2). No seizures were observed in Wistar (control) rats.

**FIGURE 2:**
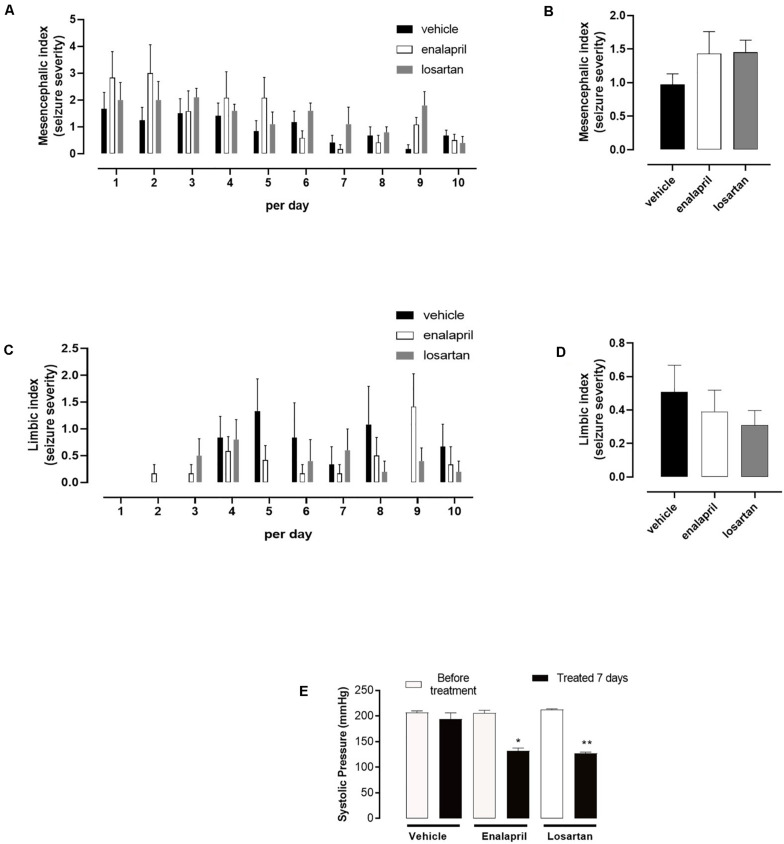
Seizure severity and systolic blood pressure (SBP) measure for spontaneous hypertensive rats (SHR) administered with vehicle (water) or enalapril (10 mg kg^– 1^) or losartan (50 mg kg^– 1^). **(A)** Severity indexes for tonic–clonic seizures (mesencephalic index) per day. **(B)** Mesencephalic indexes mean for tonic–clonic seizures. **(C)** Severity indexes for temporal lobe epilepsy seizures (limbic index) per day. **(D)** Limbic indexes mean for temporal lobe epilepsy seizures. **(E)** SBP measured in conscious SHR before treatment and on the seventh day of pharmacological treatment. **(A,C)** Data are expressed as mean ± SEM for each group (vehicle, enalapril, or losartan), per day. **(B,D)** Data are expressed as mean ± SEM for each group (vehicle, enalapril, or losartan), and analysis of variance (ANOVA) was used to compare between groups. **(E)** Data are expressed as mean ± SEM for each group (before treatment and treated 7 days for vehicle, enalapril, or losartan) and Student’s *t*-test was used to compare before treatment and treated 7 days groups. *p < 0.05. **p < 0.01. For acoustic stimulation, vehicle (*n* = 10), enalapril (*n* = 8), and losartan (*n* = 8). For SBP measure, vehicle (*n* = 10), enalapril (*n* = 8), and losartan (*n* = 8).

As expected, SBP measured prior to the first acoustic stimulus was markedly increased in SHR when compared to Wistar control (210 ± 2 vs. 107 ± 5), Figure 1E). Next, we investigated further whether a treatment with RAS inhibitors (ACE inhibitor and AT_1_ receptor antagonist) would prevent seizures in the SHR group, submitted to the same chronic acoustic stimulation protocol. As shown in Figures 2A,B, enalapril or losartan treatment did not significantly suppress the tonic–clonic seizures (mesencephalic index) in SHR, when compared to SHR treated with vehicle. Similarly, limbic seizures (limbic index, Figures 2C,D) were not affected after enalapril or losartan treatment. Despite having no effect in the seizure severity, as shown in Figure 2E, both enalapril and losartan were able to decrease blood pressure in SHR. Furthermore, Wistar rats treated with the same dose of both enalapril or losartan or vehicle had no effects in the blood pressure or seizure behavior (not shown).

## Discussion

Our study investigated the involvement of RAS in epileptic seizures behavior evoked by chronic acoustic stimulation (audiogenic kindling) in animals of the SHR strain. Although chronic treatment with the RAS inhibitors, such as ACE inhibitors and AT1 receptor antagonist, reduced blood pressure in SHR, the same treatment was not associated with reduction in epileptic seizures, as detected by the mesencephalic index (acute audiogenic seizures) and forebrain index (chronic audiogenic seizures/audiogenic kindling). This suggests that the classical systemic RAS components are not linked directly to audiogenic seizure susceptibility in animals of the SHR strain. This was in contrast to other studies, where the same treatment was able to decrease the seizure severity in the Wistar audiogenic rat (Pereira et al., 2010) strain and in SHR after kainate-induced SE (Tchekalarova et al., 2016). These comparative controversial results may be explained by the fact that epilepsy is not a specific disease. In fact, epilepsies are a heterogeneous group of disorders resulting from altered brain functions. The triggers that induce seizures in rats could involve different pathways, sometimes secondary to some pathological processes, such as hypertension and cardiovascular disturbances (Szczurkowska et al., 2019).

In the current study, we show that SHR animals subjected to audiogenic kindling developed epileptic seizures of low-level severity, when compared to those displayed by WARs (Garcia-Cairasco et al., 2017). For comparison, the highest levels found in the current study were of mesencephalic index 3 (see Figure 1), and the maximum of the mesencephalic index is 8. Moreover, while RAS inhibitors effectively lowered blood pressure in SHR, they were not able to block even low levels of audiogenic seizures in the same animals. This result was unexpected in the light of our previous study showing that the RAS inhibitors were able to reduce the severity of tonic–clonic seizures and block limbic seizures during audiogenic kindling in WARs, as well as to reduce their blood pressure levels (Pereira et al., 2010). However, it is important to highlight that SHR were selected for high blood pressure phenotype and WARs for high seizure severity phenotype. Susceptibility to seizures are in fact a comorbidity for SHR (Tchekalarova et al., 2015), and hypertension or ectopic beats are comorbidities for WARs (Garcia-Cairasco et al., 2017). In the context of our findings, it is of great interest to verify whether patients with epilepsy will improve their excitability control with blood pressure control. Conversely, it will be also important to see if controlling epileptic seizures may improve blood pressure levels/control. Such a type of interaction has been demonstrated in the scenario of epilepsy and depression (Kanner et al., 2018) and hypertension and depression (Polanka et al., 2018).

Similar to our results, it has been shown that SHR exhibited higher susceptibility to SE after the kainate (KA) model of temporal lobe epilepsy (TLE) (Tchekalarova et al., 2011) and after amygdala and piriform cortex stimulation (Greenwood et al., 1989). The treatment with AT1R antagonists did not prevent the development of SE in SHRs or WKY rats (Tchekalarova et al., 2015). It was hypothesized that non-responsiveness to treatment to AT1R antagonists may be associated with plasticity induced by hippocampal extracellular noradrenaline, serotonin, and dopamine levels. Further, the data by Atanasova et al. (2018) showed that AT1 receptor expression was increased in the amygdala of epileptic SHRs model by kainate but not of epileptic Wistar rats. In addition, long-term treatment with losartan was capable of suppressing the AT1 receptor expression in SHRs, when compared to controls. Losartan showed neuroprotection in the hippocampus and of the dentate gyrus in SHRs after kainate. However, the AT1 receptor antagonist did not exert a substantial influence on epilepsy behavioral (Tchekalarova et al., 2016).

Even though RAS inhibitors showed no effect on reducing epileptic seizures in some comorbidities models, the observation that losartan potentiates the anticonvulsant effects of other drugs could justify further investigation. Lukawski et al. (2010), for example, demonstrated that losartan potentiates the anticonvulsant effects of carbamazepine and lamotrigine in the electroshock model.

## Conclusion

In conclusion, this study demonstrates that animals of the SHR strain, phenotypically selected for hypertension, are susceptible to audiogenic seizures of low-level severity. Still, these seizures appear not to be related to the blockade of the traditional components of the RAS when induced by acoustic stimulation.

## Data Availability Statement

The original contributions presented in the study are included in the article/supplementary material, further inquiries can be directed to the corresponding author/s.

## Ethics Statement

All experiments were conducted in accordance with the National Institutes of Health Guide for the Care and Use of Laboratory Animals and were reviewed and approved by the Animal Care and Use Committee of the Ribeirão Preto Medical School, University of São Paulo.

## Author Contributions

CB and MP conducted all the experiments and wrote the manuscript. JO and NG-C provided rats from the breeding colony. KP analyzed all the results and corrected the English version. GP and JO conducted part of the experiments regarding audiogenic kindling and wrote part of the manuscript. CB, CC-N, NG-C, and MP conceived the idea of the experimental design and analyzed all the results. All authors have approved the final version of the manuscript and agreed to be accountable for all aspects of the work.

## Conflict of Interest

The authors declare that the research was conducted in the absence of any commercial or financial relationships that could be construed as a potential conflict of interest.
